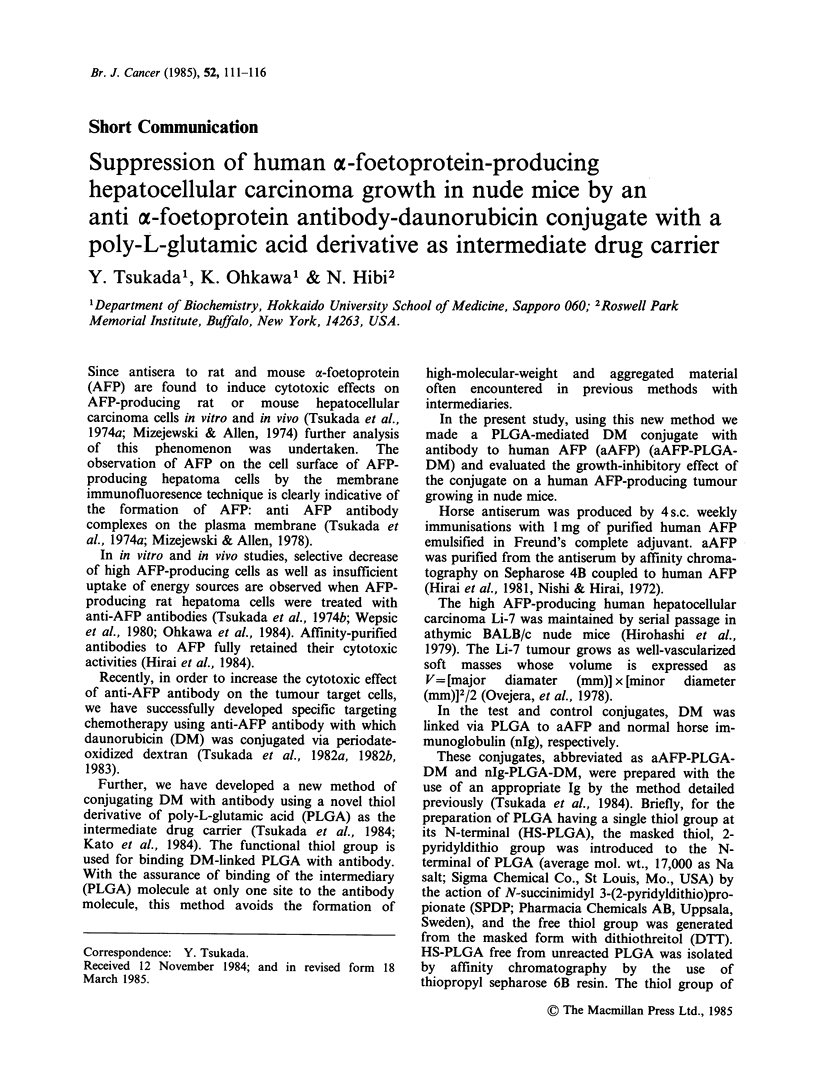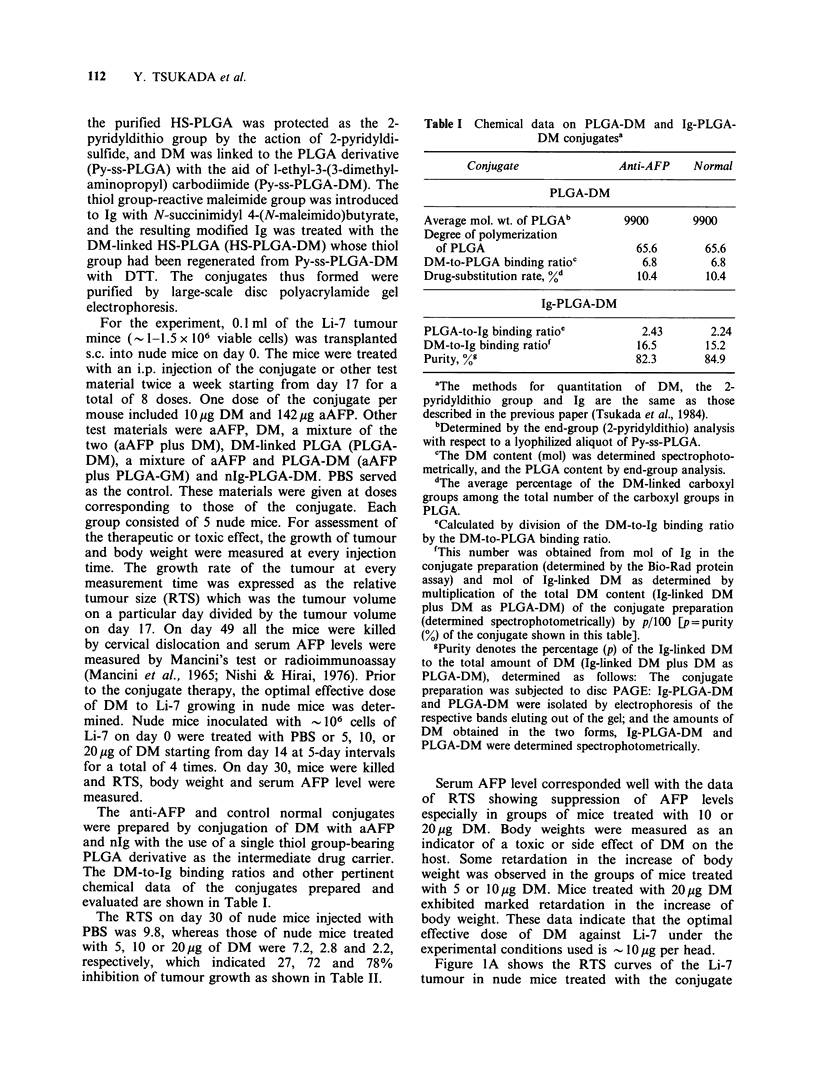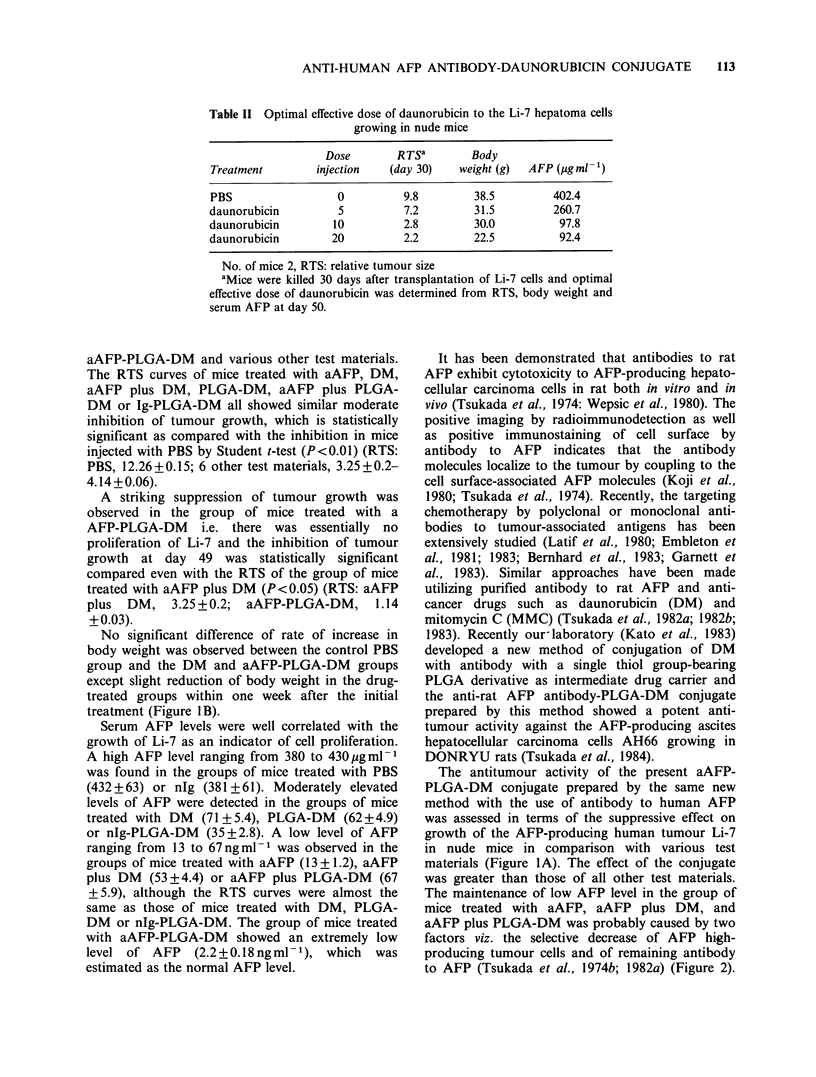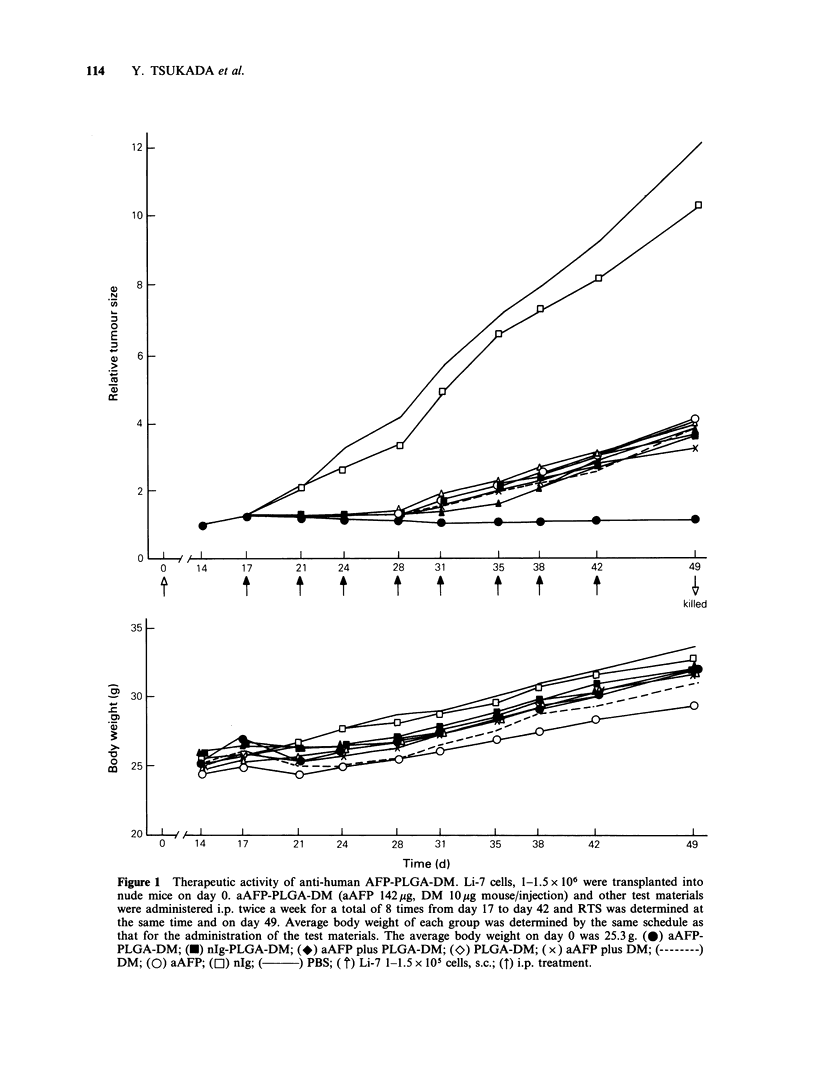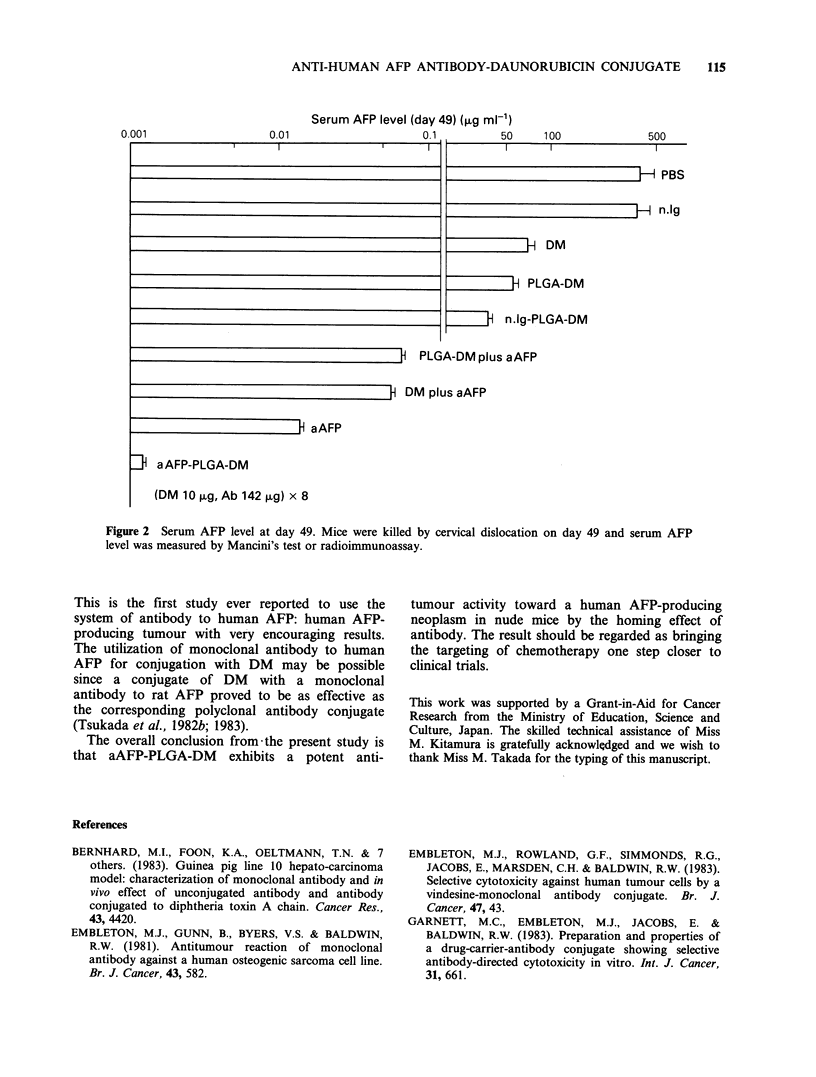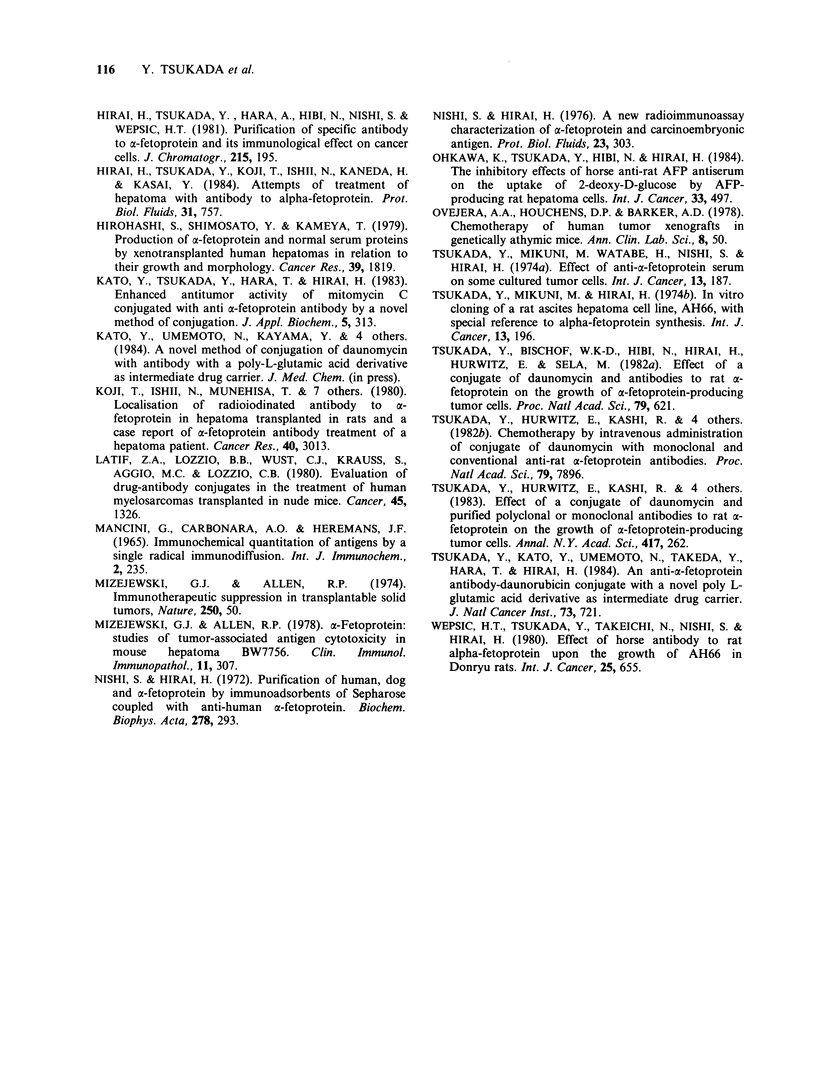# Suppression of human alpha-foetoprotein-producing hepatocellular carcinoma growth in nude mice by an anti alpha-foetoprotein antibody-daunorubicin conjugate with a poly-L-glutamic acid derivative as intermediate drug carrier.

**DOI:** 10.1038/bjc.1985.157

**Published:** 1985-07

**Authors:** Y. Tsukada, K. Ohkawa, N. Hibi


*Br. J. Cancer* (1985), **52**, 111–116

### Short Communication

### Suppression of human α-foetoprotein-producing hepatocellular carcinoma growth in nude mice by an anti α-foetoprotein antibody-daunorubicin conjugate with a poly-L-glutamic acid derivative as intermediate drug carrier

Y. Tsukada[1], K. Ohkawa[1] & N. Hibi[2]

[1]*Department of Biochemistry, Hokkaido University School of Medicine, Sapporo 060;* [2]*Roswell Park Memorial Institute, Buffalo, New York, 14263, USA.*

Since antisera to rat and mouse α-foetoprotein (AFP) are found to induce cytotoxic effects on AFP-producing rat or mouse hepatocellular carcinoma cells *in vitro* and *in vivo* (Tsukada *et al.*, 1974*a*; Mizejewski & Allen, 1974) further analysis of this phenomenon was undertaken. The observation of AFP on the cell surface of AFP-producing hepatoma cells by the membrane immunofluoresence technique is clearly indicative of the formation of AFP: anti AFP antibody complexes on the plasma membrane (Tsukada *et al.*, 1974*a*; Mizejewski & Allen, 1978).

In *in vitro* and *in vivo* studies, selective decrease of high AFP-producing cells as well as insufficient uptake of energy sources are observed when AFP-producing rat hepatoma cells were treated with anti-AFP antibodies (Tsukada *et al.*, 1974*b*; Wepsic *et al.*, 1980; Ohkawa *et al.*, 1984). Affinity-purified antibodies to AFP fully retained their cytotoxic activities (Hirai *et al.*, 1984).

Recently, in order to increase the cytotoxic effect of anti-AFP antibody on the tumour target cells, we have successfully developed specific targeting chemotherapy using anti-AFP antibody with which daunorubicin (DM) was conjugated via periodate-oxidized dextran (Tsukada *et al.*, 1982*a*, 1982*b*, 1983).

Further, we have developed a new method of conjugating DM with antibody using a novel thiol derivative of poly-L-glutamic acid (PLGA) as the intermediate drug carrier (Tsukada *et al.*, 1984; Kato *et al.*, 1984). The functional thiol group is used for binding DM-linked PLGA with antibody. With the assurance of binding of the intermediary (PLGA) molecule at only one site to the antibody molecule, this method avoids the formation of high-molecular-weight and aggregated material often encountered in previous methods with intermediaries.

In the present study, using this new method we made a PLGA-mediated DM conjugate with antibody to human AFP (aAFP) (aAFP-PLGA-DM) and evaluated the growth-inhibitory effect of the conjugate on a human AFP-producing tumour growing in nude mice.

Horse antiserum was produced by 4 s.c. weekly immunisations with 1 mg of purified human AFP emulsified in Freund's complete adjuvant. aAFP was purified from the antiserum by affinity chromatography on Sepharose 4B coupled to human AFP (Hirai *et al.*, 1981, Nishi & Hirai, 1972).

The high AFP-producing human hepatocellular carcinoma Li-7 was maintained by serial passage in athymic BALB/c nude mice (Hirohashi *et al.*, 1979). The Li-7 tumour grows as well-vascularized soft masses whose volume is expressed as $V=[\text{major diamater (mm)}]\times[\text{minor diameter (mm)}]^2/2$ (Ovejera, *et al.*, 1978).

In the test and control conjugates, DM was linked via PLGA to aAFP and normal horse immunoglobulin (nIg), respectively.

These conjugates, abbreviated as aAFP-PLGA-DM and nIg-PLGA-DM, were prepared with the use of an appropriate Ig by the method detailed previously (Tsukada *et al.*, 1984). Briefly, for the preparation of PLGA having a single thiol group at its N-terminal (HS-PLGA), the masked thiol, 2-pyridyldithio group was introduced to the N-terminal of PLGA (average mol. wt., 17,000 as Na salt; Sigma Chemical Co., St Louis, Mo., USA) by the action of *N*-succinimidyl 3-(2-pyridyldithio)propionate (SPDP; Pharmacia Chemicals AB, Uppsala, Sweden), and the free thiol group was generated from the masked form with dithiothreitol (DTT). HS-PLGA free from unreacted PLGA was isolated by affinity chromatography by the use of thiopropyl sepharose 6B resin. The thiol group of

Correspondence: Y. Tsukada.
Received 12 November 1984; and in revised form 18 March 1985.

© The Macmillan Press Ltd., 1985

the purified HS-PLGA was protected as the 2-pyridyldithio group by the action of 2-pyridyldisulfide, and DM was linked to the PLGA derivative (Py-ss-PLGA) with the aid of l-ethyl-3-(3-dimethylaminopropyl) carbodiimide (Py-ss-PLGA-DM). The thiol group-reactive maleimide group was introduced to Ig with *N*-succinimidyl 4-(*N*-maleimido)butyrate, and the resulting modified Ig was treated with the DM-linked HS-PLGA (HS-PLGA-DM) whose thiol group had been regenerated from Py-ss-PLGA-DM with DTT. The conjugates thus formed were purified by large-scale disc polyacrylamide gel electrophoresis.

For the experiment, 0.1 ml of the Li-7 tumour mince ($\sim$1–1.5 x $10^6$ viable cells) was transplanted s.c. into nude mice on day 0. The mice were treated with an i.p. injection of the conjugate or other test material twice a week starting from day 17 for a total of 8 doses. One dose of the conjugate per mouse included 10 $\mu$g DM and 142 $\mu$g aAFP. Other test materials were aAFP, DM, a mixture of the two (aAFP plus DM), DM-linked PLGA (PLGA-DM), a mixture of aAFP and PLGA-DM (aAFP plus PLGA-GM) and nIg-PLGA-DM. PBS served as the control. These materials were given at doses corresponding to those of the conjugate. Each group consisted of 5 nude mice. For assessment of the therapeutic or toxic effect, the growth of tumour and body weight were measured at every injection time. The growth rate of the tumour at every measurement time was expressed as the relative tumour size (RTS) which was the tumour volume on a particular day divided by the tumour volume on day 17. On day 49 all the mice were killed by cervical dislocation and serum AFP levels were measured by Mancini's test or radioimmunoassay (Mancini *et al.*, 1965; Nishi & Hirai, 1976). Prior to the conjugate therapy, the optimal effective dose of DM to Li-7 growing in nude mice was determined. Nude mice inoculated with $\sim 10^6$ cells of Li-7 on day 0 were treated with PBS or 5, 10, or 20 $\mu$g of DM starting from day 14 at 5-day intervals for a total of 4 times. On day 30, mice were killed and RTS, body weight and serum AFP level were measured.

The anti-AFP and control normal conjugates were prepared by conjugation of DM with aAFP and nIg with the use of a single thiol group-bearing PLGA derivative as the intermediate drug carrier. The DM-to-Ig binding ratios and other pertinent chemical data of the conjugates prepared and evaluated are shown in Table I.

The RTS on day 30 of nude mice injected with PBS was 9.8, whereas those of nude mice treated with 5, 10 or 20 $\mu$g of DM were 7.2, 2.8 and 2.2, respectively, which indicated 27, 72 and 78% inhibition of tumour growth as shown in Table II.

**Table I** Chemical data on PLGA-DM and Ig-PLGA-DM conjugates[a]

| *Conjugate* | *Anti-AFP* | *Normal* |
|---|---|---|
| PLGA-DM | | |
| Average mol. wt. of PLGA[b] | 9900 | 9900 |
| Degree of polymerization of PLGA | 65.6 | 65.6 |
| DM-to-PLGA binding ratio[c] | 6.8 | 6.8 |
| Drug-substitution rate, %[d] | 10.4 | 10.4 |
| Ig-PLGA-DM | | |
| PLGA-to-Ig binding ratio[e] | 2.43 | 2.24 |
| DM-to-Ig binding ratio[f] | 16.5 | 15.2 |
| Purity, %[g] | 82.3 | 84.9 |

[a]The methods for quantitation of DM, the 2-pyridyldithio group and Ig are the same as those described in the previous paper (Tsukada *et al.*, 1984).

[b]Determined by the end-group (2-pyridyldithio) analysis with respect to a lyophilized aliquot of Py-ss-PLGA.

[c]The DM content (mol) was determined spectrophotometrically, and the PLGA content by end-group analysis.

[d]The average percentage of the DM-linked carboxyl groups among the total number of the carboxyl groups in PLGA.

[e]Calculated by division of the DM-to-Ig binding ratio by the DM-to-PLGA binding ratio.

[f]This number was obtained from mol of Ig in the conjugate preparation (determined by the Bio-Rad protein assay) and mol of Ig-linked DM as determined by multiplication of the total DM content (Ig-linked DM plus DM as PLGA-DM) of the conjugate preparation (determined spectrophotometrically) by $p/100$ [$p$ = purity (%) of the conjugate shown in this table].

[g]Purity denotes the percentage ($p$) of the Ig-linked DM to the total amount of DM (Ig-linked DM plus DM as PLGA-DM), determined as follows: The conjugate preparation was subjected to disc PAGE: Ig-PLGA-DM and PLGA-DM were isolated by electrophoresis of the respective bands eluting out of the gel; and the amounts of DM obtained in the two forms, Ig-PLGA-DM and PLGA-DM were determined spectrophotometrically.

Serum AFP level corresponded well with the data of RTS showing suppression of AFP levels especially in groups of mice treated with 10 or 20 $\mu$g DM. Body weights were measured as an indicator of a toxic or side effect of DM on the host. Some retardation in the increase of body weight was observed in the groups of mice treated with 5 or 10 $\mu$g DM. Mice treated with 20 $\mu$g DM exhibited marked retardation in the increase of body weight. These data indicate that the optimal effective dose of DM against Li-7 under the experimental conditions used is $\sim$10 $\mu$g per head.

Figure 1A shows the RTS curves of the Li-7 tumour in nude mice treated with the conjugate

**Table II** Optimal effective dose of daunorubicin to the Li-7 hepatoma cells growing in nude mice

| *Treatment* | *Dose injection* | *RTS*[a] *(day 30)* | *Body weight (g)* | *AFP* ($\mu g\ ml^{-1}$) |
|---|---|---|---|---|
| PBS | 0 | 9.8 | 38.5 | 402.4 |
| daunorubicin | 5 | 7.2 | 31.5 | 260.7 |
| daunorubicin | 10 | 2.8 | 30.0 | 97.8 |
| daunorubicin | 20 | 2.2 | 22.5 | 92.4 |

No. of mice 2, RTS: relative tumour size
[a]Mice were killed 30 days after transplantation of Li-7 cells and optimal effective dose of daunorubicin was determined from RTS, body weight and serum AFP at day 50.

aAFP-PLGA-DM and various other test materials. The RTS curves of mice treated with aAFP, DM, aAFP plus DM, PLGA-DM, aAFP plus PLGA-DM or Ig-PLGA-DM all showed similar moderate inhibition of tumour growth, which is statistically significant as compared with the inhibition in mice injected with PBS by Student *t*-test ($P<0.01$) (RTS: PBS, 12.26±0.15; 6 other test materials, 3.25±0.2–4.14±0.06).

A striking suppression of tumour growth was observed in the group of mice treated with a AFP-PLGA-DM i.e. there was essentially no proliferation of Li-7 and the inhibition of tumour growth at day 49 was statistically significant compared even with the RTS of the group of mice treated with aAFP plus DM ($P<0.05$) (RTS: aAFP plus DM, 3.25±0.2; aAFP-PLGA-DM, 1.14 ±0.03).

No significant difference of rate of increase in body weight was observed between the control PBS group and the DM and aAFP-PLGA-DM groups except slight reduction of body weight in the drug-treated groups within one week after the initial treatment (Figure 1B).

Serum AFP levels were well correlated with the growth of Li-7 as an indicator of cell proliferation. A high AFP level ranging from 380 to 430 $\mu g\ ml^{-1}$ was found in the groups of mice treated with PBS (432±63) or nIg (381±61). Moderately elevated levels of AFP were detected in the groups of mice treated with DM (71±5.4), PLGA-DM (62±4.9) or nIg-PLGA-DM (35±2.8). A low level of AFP ranging from 13 to 67 $ng\ ml^{-1}$ was observed in the groups of mice treated with aAFP (13±1.2), aAFP plus DM (53±4.4) or aAFP plus PLGA-DM (67 ±5.9), although the RTS curves were almost the same as those of mice treated with DM, PLGA-DM or nIg-PLGA-DM. The group of mice treated with aAFP-PLGA-DM showed an extremely low level of AFP (2.2±0.18 $ng\ ml^{-1}$), which was estimated as the normal AFP level.

It has been demonstrated that antibodies to rat AFP exhibit cytotoxicity to AFP-producing hepatocellular carcinoma cells in rat both *in vitro* and *in vivo* (Tsukada *et al.*, 1974: Wepsic *et al.*, 1980). The positive imaging by radioimmunodetection as well as positive immunostaining of cell surface by antibody to AFP indicates that the antibody molecules localize to the tumour by coupling to the cell surface-associated AFP molecules (Koji *et al.*, 1980; Tsukada *et al.*, 1974). Recently, the targeting chemotherapy by polyclonal or monoclonal antibodies to tumour-associated antigens has been extensively studied (Latif *et al.*, 1980; Embleton *et al.*, 1981; 1983; Bernhard *et al.*, 1983; Garnett *et al.*, 1983). Similar approaches have been made utilizing purified antibody to rat AFP and anticancer drugs such as daunorubicin (DM) and mitomycin C (MMC) (Tsukada *et al.*, 1982*a*; 1982*b*; 1983). Recently our laboratory (Kato *et al.*, 1983) developed a new method of conjugation of DM with antibody with a single thiol group-bearing PLGA derivative as intermediate drug carrier and the anti-rat AFP antibody-PLGA-DM conjugate prepared by this method showed a potent antitumour activity against the AFP-producing ascites hepatocellular carcinoma cells AH66 growing in DONRYU rats (Tsukada *et al.*, 1984).

The antitumour activity of the present aAFP-PLGA-DM conjugate prepared by the same new method with the use of antibody to human AFP was assessed in terms of the suppressive effect on growth of the AFP-producing human tumour Li-7 in nude mice in comparison with various test materials (Figure 1A). The effect of the conjugate was greater than those of all other test materials. The maintenance of low AFP level in the group of mice treated with aAFP, aAFP plus DM, and aAFP plus PLGA-DM was probably caused by two factors *viz.* the selective decrease of AFP high-producing tumour cells and of remaining antibody to AFP (Tsukada *et al.*, 1974*b*; 1982*a*) (Figure 2).

**Figure 1** Therapeutic activity of anti-human AFP-PLGA-DM. Li-7 cells, $1–1.5 \times 10^6$ were transplanted into nude mice on day 0. aAFP-PLGA-DM (aAFP 142 $\mu$g, DM 10 $\mu$g mouse/injection) and other test materials were administered i.p. twice a week for a total of 8 times from day 17 to day 42 and RTS was determined at the same time and on day 49. Average body weight of each group was determined by the same schedule as that for the administration of the test materials. The average body weight on day 0 was 25.3 g. (●) aAFP-PLGA-DM; (■) nIg-PLGA-DM; (◆) aAFP plus PLGA-DM; (◇) PLGA-DM; (×) aAFP plus DM; (--------) DM; (○) aAFP; (□) nIg; (———) PBS; (↑) Li-7 $1–1.5 \times 10^5$ cells, s.c.; (↑) i.p. treatment.

**Figure 2** Serum AFP level at day 49. Mice were killed by cervical dislocation on day 49 and serum AFP level was measured by Mancini's test or radioimmunoassay.

This is the first study ever reported to use the system of antibody to human AFP: human AFP-producing tumour with very encouraging results. The utilization of monoclonal antibody to human AFP for conjugation with DM may be possible since a conjugate of DM with a monoclonal antibody to rat AFP proved to be as effective as the corresponding polyclonal antibody conjugate (Tsukada *et al.*, 1982*b*; 1983).

The overall conclusion from the present study is that aAFP-PLGA-DM exhibits a potent anti-tumour activity toward a human AFP-producing neoplasm in nude mice by the homing effect of antibody. The result should be regarded as bringing the targeting of chemotherapy one step closer to clinical trials.

This work was supported by a Grant-in-Aid for Cancer Research from the Ministry of Education, Science and Culture, Japan. The skilled technical assistance of Miss M. Kitamura is gratefully acknowledged and we wish to thank Miss M. Takada for the typing of this manuscript.

### References

BERNHARD, M.I., FOON, K.A., OELTMANN, T.N. & 7 others. (1983). Guinea pig line 10 hepato-carcinoma model: characterization of monoclonal antibody and *in vivo* effect of unconjugated antibody and antibody conjugated to diphtheria toxin A chain. *Cancer Res.*, **43**, 4420.

EMBLETON, M.J., GUNN, B., BYERS, V.S. & BALDWIN, R.W. (1981). Antitumour reaction of monoclonal antibody against a human osteogenic sarcoma cell line. *Br. J. Cancer*, **43**, 582.

EMBLETON, M.J., ROWLAND, G.F., SIMMONDS, R.G., JACOBS, E., MARSDEN, C.H. & BALDWIN, R.W. (1983). Selective cytotoxicity against human tumour cells by a vindesine-monoclonal antibody conjugate. *Br. J. Cancer*, **47**, 43.

GARNETT, M.C., EMBLETON, M.J., JACOBS, E. & BALDWIN, R.W. (1983). Preparation and properties of a drug-carrier-antibody conjugate showing selective antibody-directed cytotoxicity in vitro. *Int. J. Cancer*, **31**, 661.

HIRAI, H., TSUKADA, Y., HARA, A., HIBI, N., NISHI, S. & WEPSIC, H.T. (1981). Purification of specific antibody to α-fetoprotein and its immunological effect on cancer cells. *J. Chromatogr.*, **215**, 195.

HIRAI, H., TSUKADA, Y., KOJI, T., ISHII, N., KANEDA, H. & KASAI, Y. (1984). Attempts of treatment of hepatoma with antibody to alpha-fetoprotein. *Prot. Biol. Fluids*, **31**, 757.

HIROHASHI, S., SHIMOSATO, Y. & KAMEYA, T. (1979). Production of α-fetoprotein and normal serum proteins by xenotransplanted human hepatomas in relation to their growth and morphology. *Cancer Res.*, **39**, 1819.

KATO, Y., TSUKADA, Y., HARA, T. & HIRAI, H. (1983). Enhanced antitumor activity of mitomycin C conjugated with anti α-fetoprotein antibody by a novel method of conjugation. *J. Appl. Biochem.*, **5**, 313.

KATO, Y., UMEMOTO, N., KAYAMA, Y. & 4 others. (1984). A novel method of conjugation of daunomycin with antibody with a poly-L-glutamic acid derivative as intermediate drug carrier. *J. Med. Chem.* (in press).

KOJI, T., ISHII, N., MUNEHISA, T. & 7 others. (1980). Localisation of radioiodinated antibody to α-fetoprotein in hepatoma transplanted in rats and a case report of α-fetoprotein antibody treatment of a hepatoma patient. *Cancer Res.*, **40**, 3013.

LATIF, Z.A., LOZZIO, B.B., WUST, C.J., KRAUSS, S., AGGIO, M.C. & LOZZIO, C.B. (1980). Evaluation of drug-antibody conjugates in the treatment of human myelosarcomas transplanted in nude mice. *Cancer*, **45**, 1326.

MANCINI, G., CARBONARA, A.O. & HEREMANS, J.F. (1965). Immunochemical quantitation of antigens by a single radical immunodiffusion. *Int. J. Immunochem.*, **2**, 235.

MIZEJEWSKI, G.J. & ALLEN, R.P. (1974). Immunotherapeutic suppression in transplantable solid tumors, *Nature*, **250**, 50.

MIZEJEWSKI, G.J. & ALLEN, R.P. (1978). α-Fetoprotein: studies of tumor-associated antigen cytotoxicity in mouse hepatoma BW7756. *Clin. Immunol. Immunopathol.*, **11**, 307.

NISHI, S. & HIRAI, H. (1972). Purification of human, dog and α-fetoprotein by immunoadsorbents of Sepharose coupled with anti-human α-fetoprotein. *Biochem. Biophys. Acta*, **278**, 293.

NISHI, S. & HIRAI, H. (1976). A new radioimmunoassay characterization of α-fetoprotein and carcinoembryonic antigen. *Prot. Biol. Fluids*, **23**, 303.

OHKAWA, K., TSUKADA, Y., HIBI, N. & HIRAI, H. (1984). The inhibitory effects of horse anti-rat AFP antiserum on the uptake of 2-deoxy-D-glucose by AFP-producing rat hepatoma cells. *Int. J. Cancer*, **33**, 497.

OVEJERA, A.A., HOUCHENS, D.P. & BARKER, A.D. (1978). Chemotherapy of human tumor xenografts in genetically athymic mice. *Ann. Clin. Lab. Sci.*, **8**, 50.

TSUKADA, Y., MIKUNI, M. WATABE, H., NISHI, S. & HIRAI, H. (1974*a*). Effect of anti-α-fetoprotein serum on some cultured tumor cells. *Int. J. Cancer*, **13**, 187.

TSUKADA, Y., MIKUNI, M. & HIRAI, H. (1974*b*). In vitro cloning of a rat ascites hepatoma cell line, AH66, with special reference to alpha-fetoprotein synthesis. *Int. J. Cancer*, **13**, 196.

TSUKADA, Y., BISCHOF, W.K-D., HIBI, N., HIRAI, H., HURWITZ, E. & SELA, M. (1982*a*). Effect of a conjugate of daunomycin and antibodies to rat α-fetoprotein on the growth of α-fetoprotein-producing tumor cells. *Proc. Natl Acad. Sci.*, **79**, 621.

TSUKADA, Y., HURWITZ, E., KASHI, R. & 4 others. (1982*b*). Chemotherapy by intravenous administration of conjugate of daunomycin with monoclonal and conventional anti-rat α-fetoprotein antibodies. *Proc. Natl Acad. Sci.*, **79**, 7896.

TSUKADA, Y., HURWITZ, E., KASHI, R. & 4 others. (1983). Effect of a conjugate of daunomycin and purified polyclonal or monoclonal antibodies to rat α-fetoprotein on the growth of α-fetoprotein-producing tumor cells. *Annal. N.Y. Acad. Sci.*, **417**, 262.

TSUKADA, Y., KATO, Y., UMEMOTO, N., TAKEDA, Y., HARA, T. & HIRAI, H. (1984). An anti-α-fetoprotein antibody-daunorubicin conjugate with a novel poly L-glutamic acid derivative as intermediate drug carrier. *J. Natl Cancer Inst.*, **73**, 721.

WEPSIC, H.T., TSUKADA, Y., TAKEICHI, N., NISHI, S. & HIRAI, H. (1980). Effect of horse antibody to rat alpha-fetoprotein upon the growth of AH66 in Donryu rats. *Int. J. Cancer*, **25**, 655.